# Supplementary material for: Genetic algorithm-based coverage path planning for autonomous aircraft cabin cleaning by reconfigurable robot
Source: PLoS One. 2026 May 28;21(5):e0349395. doi: 10.1371/journal.pone.0349395 (PMC13218528; doi:10.1371/journal.pone.0349395)
Supplement: Attachment — Submitted filename: Detailed Response to Reviewers.docx [file pone.0349395.s003.docx]

**Editor comments:**

Dear Dr. Le,

Thank you for submitting your manuscript to PLOS ONE. After careful consideration, we feel that it has merit but does not fully meet PLOS ONE’s publication criteria as it currently stands. Therefore, we invite you to submit a revised version of the manuscript that addresses the points raised during the review process.

More specifically, in the revised version of the paper please clarify and justify the algorithmic design (Stage-1 detection, waypoint generation, cost weights, feasibility of GA paths), report full final coverage after Stage-2, and strengthen the experimental validation (fair multi-run comparisons, scalability beyond 50 seats, and realistic motion/coverage modeling). In addition, please explain why only up to 50 seats are tested and ensure formatting, figures, and references are fully consistent.

Please submit your revised manuscript by Mar 01 2026 11:59PM. If you will need more time than this to complete your revisions, please reply to this message or contact the journal office at [plosone@plos.org](mailto:plosone@plos.org). When you're ready to submit your revision, log on to <https://www.editorialmanager.com/pone/> and select the 'Submissions Needing Revision' folder to locate your manuscript file.

Please include the following items when submitting your revised manuscript:

- A letter that responds to each point raised by the academic editor and reviewer(s). You should upload this letter as a separate file labeled 'Response to Reviewers'.
- A marked-up copy of your manuscript that highlights changes made to the original version. You should upload this as a separate file labeled 'Revised Manuscript with Track Changes'.
- An unmarked version of your revised paper without tracked changes. You should upload this as a separate file labeled 'Manuscript'.

If you would like to make changes to your financial disclosure, please include your updated statement in your cover letter. Guidelines for resubmitting your figure files are available below the reviewer comments at the end of this letter.

If applicable, we recommend that you deposit your laboratory protocols in protocols.io to enhance the reproducibility of your results. Protocols.io assigns your protocol its own identifier (DOI) so that it can be cited independently in the future. For instructions see: [https://journals.plos.org/plosone/s/submission-guidelines#loc-laboratory-protocols](https://track.editorialmanager.com/CL0/https:%2F%2Fjournals.plos.org%2Fplosone%2Fs%2Fsubmission-guidelines%23loc-laboratory-protocols/1/010f019bc0ad8dd6-24849a36-9741-44a9-8780-2c2ced4e133d-000000/01oYv4touRv9D07_pxvVLdJMpXrOYrtoRGqPc86DKtA=246). Additionally, PLOS ONE offers an option for publishing peer-reviewed Lab Protocol articles, which describe protocols hosted on protocols.io. Read more information on sharing protocols at [https://plos.org/protocols?utm_medium=editorial-email&utm_source=authorletters&utm_campaign=protocols](https://track.editorialmanager.com/CL0/https:%2F%2Fplos.org%2Fprotocols%3Futm_medium=editorial-email%26utm_source=authorletters%26utm_campaign=protocols/1/010f019bc0ad8dd6-24849a36-9741-44a9-8780-2c2ced4e133d-000000/_2xi2U-_k9cXA8M-dV9WIRTCVLs32vg1aGqVdf-usrY=246).

We look forward to receiving your revised manuscript.

Kind regards,

Camelia Delcea

Academic Editor

PLOS One

Journal Requirements:

When submitting your revision, we need you to address these additional requirements.

1.Please ensure that your manuscript meets PLOS ONE's style requirements, including those for file naming. The PLOS ONE style templates can be found at

<https://journals.plos.org/plosone/s/file?id=wjVg/PLOSOne_formatting_sample_main_body.pdf> and

[https://journals.plos.org/plosone/s/file?id=ba62/PLOSOne_formatting_sample_title_authors_affiliations.pdf](https://track.editorialmanager.com/CL0/https:%2F%2Fjournals.plos.org%2Fplosone%2Fs%2Ffile%3Fid=ba62%2FPLOSOne_formatting_sample_title_authors_affiliations.pdf/1/010f019bc0ad8dd6-24849a36-9741-44a9-8780-2c2ced4e133d-000000/qFNVAm1J761FBwpuBYXz3OJAIwm3lv8P8FGUif-toes=246)

2. Please note that PLOS One has specific guidelines on code sharing for submissions in which author-generated code underpins the findings in the manuscript. In these cases, we expect all author-generated code to be made available without restrictions upon publication of the work. Please review our guidelines at <https://journals.plos.org/plosone/s/materials-and-software-sharing#loc-sharing-code> and ensure that your code is shared in a way that follows best practice and facilitates reproducibility and reuse.

3. We note that your Data Availability Statement is currently as follows: All relevant data are within the manuscript and in Supporting Information files.

Please confirm at this time whether or not your submission contains all raw data required to replicate the results of your study. Authors must share the “minimal data set” for their submission. PLOS defines the minimal data set to consist of the data required to replicate all study findings reported in the article, as well as related metadata and methods (<https://journals.plos.org/plosone/s/data-availability#loc-minimal-data-set-definition>).

For example, authors should submit the following data:

- The values behind the means, standard deviations and other measures reported;

- The values used to build graphs;

- The points extracted from images for analysis.

Authors do not need to submit their entire data set if only a portion of the data was used in the reported study.

If your submission does not contain these data, please either upload them as Supporting Information files or deposit them to a stable, public repository and provide us with the relevant URLs, DOIs, or accession numbers. For a list of recommended repositories, please see

<https://journals.plos.org/plosone/s/recommended-repositories>.

If there are ethical or legal restrictions on sharing a de-identified data set, please explain them in detail (e.g., data contain potentially sensitive information, data are owned by a third-party organization, etc.) and who has imposed them (e.g., an ethics committee). Please also provide contact information for a data access committee, ethics committee, or other institutional body to which data requests may be sent. If data are owned by a third party, please indicate how others may request data access.

4. We note that Figures 1,2 and 5 in your submission contain copyrighted images. All PLOS content is published under the Creative Commons Attribution License (CC BY 4.0), which means that the manuscript, images, and Supporting Information files will be freely available online, and any third party is permitted to access, download, copy, distribute, and use these materials in any way, even commercially, with proper attribution. For more information, see our copyright guidelines: <http://journals.plos.org/plosone/s/licenses-and-copyright>.

 We require you to either (1) present written permission from the copyright holder to publish these figures specifically under the CC BY 4.0 license, or (2) remove the figures from your submission:

 1. You may seek permission from the original copyright holder of Figures 1,2 and 5 to publish the content specifically under the CC BY 4.0 license.

 We recommend that you contact the original copyright holder with the Content Permission Form (<http://journals.plos.org/plosone/s/file?id=7c09/content-permission-form.pdf>) and the following text:

“I request permission for the open-access journal PLOS ONE to publish XXX under the Creative Commons Attribution License (CCAL) CC BY 4.0 (<http://creativecommons.org/licenses/by/4.0/>). Please be aware that this license allows unrestricted use and distribution, even commercially, by third parties. Please reply and provide explicit written permission to publish XXX under a CC BY license and complete the attached form.”

 Please upload the completed Content Permission Form or other proof of granted permissions as an "Other" file with your submission.

 In the figure caption of the copyrighted figure, please include the following text: “Reprinted from [ref] under a CC BY license, with permission from [name of publisher], original copyright [original copyright year].”

 2. If you are unable to obtain permission from the original copyright holder to publish these figures under the CC BY 4.0 license or if the copyright holder’s requirements are incompatible with the CC BY 4.0 license, please either i) remove the figure or ii) supply a replacement figure that complies with the CC BY 4.0 license. Please check copyright information on all replacement figures and update the figure caption with source information. If applicable, please specify in the figure caption text when a figure is similar but not identical to the original image and is therefore for illustrative purposes only.

5. If the reviewer comments include a recommendation to cite specific previously published works, please review and evaluate these publications to determine whether they are relevant and should be cited. There is no requirement to cite these works unless the editor has indicated otherwise. 

**Author response for editor:**

Thank you for allowing us to revise the manuscripts. We have addressed the point-to-point comments of the editors and reviewers. Please find the revised manuscript and response for reviewers for your details.

**Reviewer comments:**

**Reviewer #1:**

**Reviewer #1 Point1:** There are above 200 seats in the aircraft cabin of Boeing 777-300ER and Airbus A321-200 respectively, but why the maximum "Numbers of Target Seats" is 50 in the table 9 and table 10? Please explain it briefly in the relative sections of this manuscript.

**Author response:** Thank you for your comment.

Although the full aircraft cabin contains more than 200 seats, the number of target seats optimized in Stage 2 small configurable robot footprint does not correspond to the total number of physical seats, but rather to the number of effective seat-level waypoints generated after Stage by large configurable robot foot print. Stage 1 performs a global sweeping coverage, during which only a subset of seats or under-seat regions are identified as skipped or hard-to-access. These skipped regions are then converted into target waypoints for Stage 2 optimization.

In the tested cabin layouts, the maximum number of such skipped or deferred regions stage 1 is up to 100 random waypoints. This number reflects the realitic case number of uncovered regions requiring revisitation. As demonstrated by the additional 100-seat experiments added in the revised manuscript, the proposed optimization approach scales beyond this value when denser waypoint distributions are considered.

**Author action:** We updated the manuscript by:

- Added sentences in Section I Introduction from line 95 to line 96 to state that complete coverage is evaluated at the two-stage framework level, not at Stage 1 alone.

In this work, complete coverage is evaluated at the level of the integrated two-stage framework, rather than at the individual stage level.

- Added sentences in Section 4.2 – Stage 1: Structured Coverage with Spiral and Zigzag Patterns from line 377 to line 381 to state that Stage 1 is designed for rapid partial coverage, prioritizing efficiency over completeness. Explicitly stated that uncovered regions are intentionally deferred to Stage 2.

Stage 1 is intentionally designed as a rapid and conservative sweeping process under the robot’s full configuration and therefore does not aim to achieve complete coverage on its own. Regions that are not covered due to accessibility constraints or conservative decisions are explicitly recorded and deferred to Stage 2 for guaranteed revisitation.

- Added sentences in Section 4.1 Overview of the Two-Stage CPP Framework 3 from line 364 to line 370 to make a statement explaining that the final coverage rate after Stage 2 reaches 100% of all reachable target regions, as confirmed by the successful visitation of all waypoints in the 30-, 50-, and 100-seat experiments.

A half-size robot is deployed to revisit waypoints identified in Stage 1 as skipped or obstructed. These waypoints visting order are optimized using a Genetic Algorithm (GA)-based planner, solving a partial-CCPP problem that resembles the Traveling Salesman Problem (TSP) guaranteeing deterministic revisitation for CCPP in Stage 2. Accordingly, the term CCPP refers to the proposed two-stage framework as a whole, rather than to the partial sweeping behavior of Stage 1 in isolation.

- Modified Figure 15 and 16 for aircraft cabin with 30, 50 and 100 randomly distributed skipped seats and the corresponding optimal path generated by the proposed method with different starting and ending locations.
- Modified Table 6, Table 8, Table 12 and 13 to add 100 skipped seats senario for path length and energy comparision.

**Reviewer #1 Point2**: Please maintain consistency in the text font, size, and formatting of the images and tables throughout the manuscript.

**Author response:** Thank you for the comment.

In the revised manuscript, we have carefully reviewed the entire document to ensure uniform font type, font size, and formatting across all text, figures, and tables.

**Author action:** We updated the manuscript by:

- Reformatted figures to ensure consistent font size, label style, and color usage.
- Standardized table captions, column headings, and numerical formatting.
- Ensured uniform text font and size throughout the manuscript in accordance with the journal template.

**Reviewer #1 Point3**. Please maintain consistency in the reference format, such as 1) DOI number, either all references are labeled or none are labeled, unless some references do not have a DOI number. 2) The capitalization, full spelling/simplified spelling, etc. of the author's name.

**Author response:** Thank you for the comment.

In the revised manuscript, all references have been thoroughly reviewed and corrected to ensure consistent formatting. DOI are now not included for all references. Author names have been standardized in terms of capitalization, spelling, and abbreviation format, and journal titles have been consistently presented according to the journal’s reference style.

**Author action:** We updated the manuscript by:

- Standardized DOI does not include across the entire reference list.
- Corrected author name capitalization and spelling inconsistencies.
- Ensured consistent formatting of journal names, volume/issue numbers, and page ranges

**Reviewer #2:**

**Reviewer #2 Point1:** What triggers the skipping of seat rows in Stage 1? How exactly are “hard-to-access seat rows” detected and determined? What are the thresholds, sensor types, and false positive rates? If false positives cause missed scans, Stage 2 cannot compensate.

**Author response:** Thank you for your comment.

The primary focus of this work is on coverage path planning and task-level optimization, rather than perception or sensor-level decision making. Consequently, specific sensor types, detection thresholds, and false-positive rates are not explicitly modeled in the present study. Instead, Stage 1 is treated as a conservative coverage process in which potentially inaccessible or uncertain regions are intentionally deferred.

To ensure that such skipped regions do not result in permanent coverage loss, all skipped seats are explicitly recorded and passed to Stage 2 large configurable robot footprint as mandatory waypoints. Stage 2 then revisits these regions using a reconfigured robot and a global optimization strategy. This design guarantees that regions skipped in Stage 1 are deterministically recovered in Stage 2, provided they are physically reachable by the reconfigurable robot proposed in the paper framework.

Nevertheless, we fully agree with the reviewer that incorporating onboard sensing uncertainty, perception noise, and real-time environmental changes would represent a meaningful and technically challenging extension of this work in the future.

**Author action:** We updated the manuscript by:

To address this comment, we have **clarified the detection logic and assumptions of Stage 1** in the revised manuscript as follows:

- Add the sententes in Section 4.1 Overview of the Two-Stage CPP Framework from line 356 to line 363 to state that hard-to-access seat rows are determined based on geometric clearance constraints relative to the robot’s full configuration, rather than sensor-based detection. Explicitly clarified that Stage 1 operates on a static reconstructed cabin layout, eliminating false positives due to perception noise.
- Added sentences in Section 4.1 Overview of the Two-Stage CPP Framework from line 364 to line 370 to make a statement explaining that all skipped seat rows are logged and transformed into waypoints, guaranteeing deterministic revisitation in Stage 2. And the perception uncertainty and sensor noise are outside the scope of this work.

**Reviewer #2 Point2:** How is the waypoint set W generated? Is one point assigned per row? Or are multiple points used to cover the area beneath seats? How are waypoints mapped to the actual coverage area?

**Author response:** Thank you for your comment regarding the construction of the waypoint set $W$.

In the proposed framework, the waypoint set $W$is generated based on the skipped coverage regions identified during Stage 1. While a simplified representation may associate a single waypoint with an entire seat row, the framework is not restricted to one waypoint per row. Instead, multiple waypoints can be generated within the same row, with each waypoint corresponding to an individual seat or under-seat region requiring coverage.

Specifically, each waypoint represents the centroid of a local coverage region beneath a seat, rather than a geometric abstraction of an entire row. The mapping between waypoints and coverage regions is therefore one-to-one, where visiting a waypoint implies coverage of the corresponding under-seat area within the reconfigurable robot’s effective cleaning footprint.

The flexibility of this waypoint definition is reflected in the revised experiments, where Stage 2 optimizes paths extend to 100 individual seat-based waypoints distributed across the cabin. These results demonstrate that the proposed framework supports multiple waypoints per row and scales effectively with increasing waypoint density to the actual coverage area.

**Author action:** We updated the manuscript by:

- Added sentences in Section 4.3 – Genetic Algorithm-Based Reconfigurable Path Optimization for Process C (Stage 2) from line 429 to line 442 to make a explicit statement that the waypoint set $W$may contain multiple waypoints within the same seat row.
- Added sentences in Section 4.6 – Experimental Results from line 580 line 582 to update the description of Stage-2 experiments to emphasize this section presents the experimental results for Process C of the proposed CCPP framework, where the cleaning robot is required to visit between 10 and 100 randomly distributed seat locations identified as requiring cleaning.

**Reviewer #2 Point3:** How are γ1, γ2, and γ3 determined in the cost function (8)? Has sensitivity analysis been conducted? Does increasing γ3 cause the algorithm to favor fewer reconstructions at the expense of coverage?

**Author response:** Thank you for your comment.

In the proposed framework, the weighting coefficients $\gamma_{1}$, $\gamma_{2}$, and $\gamma_{3}$are empirically determined through preliminary tuning experiments to balance three competing objectives: travel distance, motion-related energy expenditure, and morphology reconfiguration cost. The tuning process aims to achieve stable convergence and reasonable trade-offs among these objectives under typical aircraft cabin layouts.

As the primary focus is on demonstrating the feasibility and scalability of the proposed two-stage coverage framework rather than parameter optimization, The robustness of the selected weights is indirectly supported by consistent algorithmic behavior observed across experiments with increasing problem sizes, including the 30-, 50-, and 100-seat scenarios.

Regarding the reviewer’s concern about $\gamma_{3}$, increasing this weight does indeed bias the optimization toward solutions with fewer morphology reconfigurations. However, this does not compromise coverage completeness, as coverage is enforced structurally by the waypoint-based formulation: all waypoints must be visited exactly once regardless of the relative weighting of cost terms. Therefore, a higher $\gamma_{3}$may affect the *order* in which waypoints are visited and the frequency of reconfiguration events, but it cannot cause uncovered regions or skipped waypoints.

We agree that a systematic sensitivity analysis exploring the influence of different weighting combinations would be a valuable extension of this work and have explicitly identified this as a direction for future research.

**Author action:** We updated the manuscript by:

To address this comment, we have clarified the role and tuning of the cost-function weights in the revised manuscript as follows:

- Added sentences in Section 4.3 – Genetic Algorithm-Based Reconfigurable Path Optimization for Process C (Stage 2) from line 467 to line 473 to add an explanation stating that $\gamma_{1}$, $\gamma_{2}$, and $\gamma_{3}$are empirically tuned through preliminary trials to balance distance, energy, and reconfiguration costs. Clarified that coverage completeness is independent of the weight values, as all waypoints must be visited by construction. And $\gamma_{3}$biases the optimization toward solutions with fewer morphology reconfigurations.
- Added sentences in Section 6 – Conclusion and Future Work from line 830 to line 832 to make a remark acknowledging that a formal sensitivity analysis of the weighting parameters is not included and identifying it as an important topic for future investigation.

**Reviewer #2 Point 4:** Emove uses a linear distance model (α·d/β·d), yet robot energy consumption heavily depends on turning, acceleration/deceleration, ground friction, and course correction in narrow passages. Why are these factors neglected? Is an upper bound on error provided?

**Author response:** Thank you for your comment.

In the proposed framework, the energy term $E_{\text{move}}$is modeled as a linear function of travel distance to maintain computational efficiency and enable fair comparison across different optimization algorithms. The primary objective of this work is to evaluate relative global path planning performance and scalability rather than to provide a high-fidelity physical energy model. Incorporating detailed dynamics such as turning effort, acceleration/deceleration, frictional losses, and corrective maneuvers in narrow passages would significantly increase model complexity and require robot-specific parameters that are beyond the scope of the current planning-focused study.

Nevertheless, we acknowledge that these neglected factors do influence real energy consumption. In practice, their effects are implicitly averaged into the motion coefficients $\alpha$and $\beta$, which are chosen to reflect typical operational conditions. As a result, the energy values reported in this paper should be interpreted as relative performance indicators rather than exact physical energy estimates.

Regarding the reviewer’s question on error bounds, we note that no explicit upper bound on the energy estimation error is provided, as this would require a detailed dynamic model and empirical validation on physical hardware. We agree that deriving such bounds would be valuable, and we have identified this as a direction for future work.

**Author action:** We updated the manuscript by:

- Added sentences Section 4.3 – Genetic Algorithm-Based Reconfigurable Path Optimization for Process C (Stage 2) from line 464 to line 466 to to add an explanation stating that $E_{\text{move}}$is modeled as a distance-based approximation to preserve computational tractability and ensure consistent comparison among algorithms
- Added sentences Section 5 Modeling Assumptions and Limitations from line 732 to line 734 to add an explanation to clarify that turning, acceleration/deceleration, friction, and local corrections are not explicitly modeled, and that their effects are implicitly captured in the motion coefficients.
- Added sentences in Section 6 High-Fidelity Energy Modeling and Parameter Sensitivity from line 826 to line 832 to mention that the distance-based energy approximation adopted in this work enables efficient algorithm comparison but does not explicitly capture dynamic effects such as turning effort, acceleration, friction, or corrective maneuvers.

**Reviewer #2 Point5:** Stage 1 coverage is only 81–86%. What is the final coverage rate after Stage 2? Does it meet the definition of “complete coverage”? If not, is the CCPP naming accurate?

**Author response:** Thank you for your comment.

In the proposed framework, Stage 1 covering the map by large configurable robot foot print is intentionally designed as a fast, conservative sweeping process to maximize efficiency under the robot’s full configuration. As a result, partial coverage (81–86%) is expected at this stage, particularly in narrow or obstructed under-seat regions. Importantly, Stage 1 is not intended to achieve complete coverage on its own.

Complete coverage is achieved at the framework level through the integration of Stage 2 covering the map by smaler configurable footprint. All regions skipped during Stage 1 are explicitly recorded and transformed into mandatory waypoints, which are then revisited in Stage 2 using a reconfigured robot and a global optimization strategy. By construction, the Stage-2 planner enforces visitation of all waypoints exactly once, ensuring that all reachable skipped regions are covered.

The extended experimental results, including 30-, 50-, and 100-seat scenarios, demonstrate that all generated waypoints are successfully visited in Stage 2, resulting in full coverage of all reachable target regions. Accordingly, the use of the term Complete Coverage Path Planning (CCPP) is accurate when referring to the two-stage framework as a whole, rather than to Stage 1 in isolation.

**Author action:** We updated the manuscript by:

- Added sentences in Section I Introduction from line 95 to line 96 to state that complete coverage is evaluated at the two-stage framework level, not at Stage 1 alone.
- Added sentences in Section 4.2 – Stage 1: Structured Coverage with Spiral and Zigzag Patterns from line 377 to line 381 to state that Stage 1 is designed for rapid partial coverage, prioritizing efficiency over completeness. Explicitly stated that uncovered regions are intentionally deferred to Stage 2.
- Added sentences in Section 4.1 Overview of the Two-Stage CPP Framework 3 from line 364 to line 370 to make a statement explaining that the final coverage rate after Stage 2 reaches 100% of all reachable target regions, as confirmed by the successful visitation of all waypoints in the 30-, 50-, and 100-seat experiments.
- Modified Figure 15 and 16 for aircraft cabin with 30, 50 and 100 randomly distributed skipped seats and the corresponding optimal path generated by the proposed method with different starting and ending locations.
- Modified Table 6, Table 8, Table 12 and 13 to add 100 skipped seats senario for path length and energy comparision.

**Reviewer #2 Point6:** Is the GA vs. ACO/DE/PSO comparison fair? Were all algorithms evaluated under the same computational budget (time/iterations/function evaluations)? Were results repeated ≥30 times to provide mean ± standard deviation with significance testing?

**Author response:** Thank you for your comment.

All optimization algorithms considered in this study—GA, ACO, DE, PSO, and the greedy baseline—were evaluated under identical problem settings, including the same cabin layout, waypoint sets, cost function, and termination criteria.

For each experimental scenario, the reported results correspond to the average performance over multiple independent runs of 30 times with different random initializations. The primary objective of this comparative study is to assess relative performance trends and scalability across algorithms rather than to conduct exhaustive statistical benchmarking.

Nevertheless, the robustness of the observed performance trends is supported by their consistency across increasing problem sizes, including the newly added 30-, 50-, and 100-seat scenarios. In all cases, the proposed GA-based approach consistently outperforms the competing methods in terms of path length and estimated energy consumption.

We agree that a more extensive statistical analysis, including larger numbers of repeated trials and formal significance testing, would further strengthen the comparison, and we have identified this as an important direction for future work.

.

**Author action:** We updated the manuscript by:

- Addem sentences in Section 4.6 Results for Process C using GA TSP Optimization-based Algorithms from line 627 to line 637 to make an explicit statement confirming that all algorithms are evaluated under the same computational budget, using identical iteration limits and cost-function evaluations for each scenario.
- Added sentences in Section 4.6 Results for Process C using GA TSP Optimization-based Algorithms from line 637 to line 643 to state that reported values represent average results over multiple independent runs of 30 times. Emphasized that the comparison focuses on relative performance trends across algorithms and problem sizes experiments.
- Modified Figure 15 and 16 for aircraft cabin with 30, 50 and 100 randomly distributed skipped seats and the corresponding optimal path generated by the proposed method with different starting and ending locations.
- Modified Table 6, Table 8, Table 12 and 13 to add 100 skipped seats senario for path length and energy comparision.

**Reviewer #2 Point7:** Table 9 only shows results for 50 target points, but the text mentions 10–100 seats. Where are the results for 100 seats? Does the algorithm degrade at larger scales?

**Author response:** Thank you for your comment.

In the revised manuscript, we have extended the experimental evaluation to explicitly include the 100-seat scenario. The corresponding results for both total path length and estimated energy consumption are now reported in Tables 9 and 10, respectively. In addition, a new composite figure illustrating GA-optimized trajectories for 30, 50, and 100 target seats has been introduced to visually demonstrate the behavior of the algorithm as the number of waypoints increases.

The newly added results show that the proposed GA-based optimization does not exhibit performance degradation at larger scales. While the absolute path length and energy consumption naturally increase with the number of target seats, the GA consistently outperforms the comparison methods (ACO, DE, PSO, and greedy search) and maintains stable convergence behavior. These trends indicate that the algorithm scales effectively with increasing waypoint density and problem size.

**Author action:** We updated the manuscript by:

- Added sentences in Section 4.6 Results for Process C using GA TSP Optimization-based Algorithms from line 639 to line 643 to add the results for the 100-seat scenario to complement the existing 10–50 seat experiments and update the discussion to emphasize scalability and to clarify that the algorithm maintains consistent performance trends as the number of target seats increases.
- Added sentences to Section 4.6 (Results for Process C using GA–TSP Optimization-Based Algorithms), lines 661–688, to present GA-optimized paths for 30, 50, and 100 target seats using a fixed start/end waypoint and spatially distributed target seats. The results demonstrate consistent performance trends with no observable degradation as problem size increases within the tested scales.
- Modified Figure 15 and 16 for aircraft cabin with 30, 50 and 100 randomly distributed skpped seats and the corresponding optimal path generated by the proposed method with different starting and ending locations.
- Modified Table 6, Table 8, Table 12 and 13 to add 100 skipped seats senario for path length and energy comparision.

**Reviewer #2 Point8:** GA uses ERX crossover with swap/toggle mutation: Could this generate infeasible paths (collisions/unreachable turns/violating narrow aisle constraints)? How is feasibility ensured?

**Author response:** Thank you for your comment.

In the proposed framework, the GA is applied at the waypoint sequencing level, where each individual represents a permutation of prevalidated waypoints rather than continuous robot motions. All waypoints are generated from collision-free regions identified on the reconstructed cabin map and are reachable by the robot in its small reconfigured mode. Consequently, the GA operators (ERX crossover and swap/toggle mutation) only reorder existing feasible waypoints and do not modify their spatial locations.

As a result, infeasibilities such as collisions, unreachable turns, or violations of narrow aisle constraints cannot be introduced by the genetic operators themselves, since these constraints are already enforced during waypoint generation and robot configuration selection. The GA optimizes only the visiting order of waypoints, while the underlying motion execution between consecutive waypoints relies on the robot’s validated kinematic and geometric feasibility.

Furthermore, the successful generation of closed-loop trajectories for increasingly dense waypoint sets, including the newly added 30-, 50-, and 100-seat scenarios, provides empirical evidence that the optimization process consistently yields feasible solutions without violating cabin constraints.

.

**Author action:** We updated the manuscript by:

- Added sentences in Section 4.3 – Stage 2: Waypoint-Based Optimization from line 652 to line 660 to add an explicit explanation stating that the GA operates on prevalidated, collision-free waypoints, and that genetic operators only alter waypoint orderings and to sate that feasibility with respect to collisions, kinematic limits, and narrow aisle constraints is enforced prior to optimization, during waypoint generation and robot configuration selection.
- Added sentences in 6.6 High-Fidelity Energy Modeling and Parameter Sensitivity from line 842 to line 846 to add a clarification noting that motion-level collision avoidance is handled implicitly by the waypoint formulation, while continuous trajectory validation is left for future execution-level integration.
- Modified Figure 15 and 16 for aircraft cabin with 30, 50 and 100 randomly distributed red seats and the corresponding optimal path generated by the proposed method with different starting and ending locations.

**Reviewer #2 Point 9:** Aircraft cleaning efficiency depends not only on path planning but also on cleaning coverage width, cleaning mechanisms, and cleaning effectiveness verification. Does the coverage definition in the paper account for cleaning width/overlap rate/edge missed scans?

**Author response:** Thank you for your comment.

In the present study, coverage is defined at the planning and geometric traversal level, focusing on whether the robot physically reaches and traverses all target regions identified for cleaning. The proposed framework addresses the coverage path planning (CPP) problem, rather than the detailed modeling of cleaning mechanisms or post-cleaning effectiveness verification.

As such, factors including cleaning coverage width, overlap rate, edge effects, suction efficiency, or sterilization effectiveness are not explicitly modeled in the current formulation. Instead, it is assumed that once the robot traverses a target region, the corresponding under-seat area falls within the effective cleaning footprint of the deployed mechanism. This abstraction is commonly adopted in CPP literature to decouple motion planning from mechanism-specific performance characteristics.

We fully agree that incorporating cleaning width, overlap constraints, and coverage verification would be necessary for deployment-ready systems. Importantly, the proposed waypoint-based formulation is compatible with such extensions, as coverage width and overlap requirements can be incorporated by refining the waypoint generation process or by introducing additional constraints on waypoint spacing. These aspects are therefore identified as promising directions for future work.

**Author action:** We updated the manuscript by:

- Added sentences in Section 5.2 – Modeling Assumptions and Limitations from line 739 to line to 741 to add an explicit clarification that coverage is defined in terms of geometric traversal of target regions, rather than cleaning effectiveness or verification metrics.
- Added sentences in in Section 5.2 – Modeling Assumptions and Limitations from line 741 to line 745 to add a statement explaining that cleaning width, overlap rate, and edge effects are not explicitly modeled, and that coverage is evaluated based on waypoint visitation.
- Added sentences in 6.5 Mechanism-Aware Coverage and Integrated Sterilization from line 816 to line 824 to add a paragraph identifying mechanism-aware coverage modeling and effectiveness verification as important extensions for future research.

**Reviewer #2 Point10:** If cabin conditions change due to cleaning staff movement, debris shifts, or reclined seats, this alters the map and navigability. Does the framework support online replanning? Or is it entirely offline? If online, how are complexity and latency guaranteed?

**Author response:** Thank you for your comment.

The proposed framework is primarily designed as an offline planning approach, operating on a reconstructed cabin map that represents a static snapshot of the environment. The focus of this work is on coverage path planning and task-level optimization, rather than real-time perception-driven replanning. As such, dynamic changes caused by cleaning staff movement, debris shifts, or seat recline are not explicitly modeled in the current implementation.

Nevertheless, the framework is conceptually compatible with online replanning extensions. In particular, the waypoint-based formulation of Stage 2 enables newly detected or reclassified regions to be appended to the waypoint set and locally re-optimized without recomputing the entire global plan. This structure allows partial replanning in response to environmental changes, should real-time perception be integrated.

Guaranteeing latency and computational complexity for full online replanning would require explicit constraints on update frequency, problem size, and solver termination conditions, which are beyond the scope of this study. We therefore position online replanning under dynamic conditions as a promising direction for future research, building upon the planning foundation established in this work.

**Author action:** We updated the manuscript by:

- Added sentences in 6.4 Online Replanning under Dynamic Cabin Conditions from line 806 to line 814 to state clarifying that the proposed framework operates on a static cabin map and is evaluated in an offline planning context that guaranteeing computational latency and complexity would require additional mechanisms beyond the current scope.

**Reviewer #2 Point11:** The paper notes a “lack of integrated sterilization capabilities.” Does the current method only apply to vacuuming? If UV/spray disinfection is added, coverage and safety constraints (obstruction, dosage) will significantly alter CPP design. Is the framework extensible?

**Author response:** Thank you for your comment.

In the current study, the framework is evaluated under the assumption of a vacuum-based cleaning mechanism, which motivates the use of geometric coverage and traversal-based definitions. Integrated sterilization mechanisms such as UV irradiation or spray-based disinfection are not explicitly modeled, and therefore safety constraints related to exposure dosage, line-of-sight obstruction, or chemical dispersion are outside the scope of the present work.

Nevertheless, the proposed framework is not restricted to vacuuming applications. The two-stage structure and waypoint-based formulation are designed to be mechanism-agnostic at the planning level. If UV or spray-based sterilization is introduced, additional constraints—such as minimum exposure time, visibility requirements, safety distances from humans, or overlap redundancy—can be incorporated by modifying the waypoint generation rules and augmenting the cost function with task-specific penalty terms.

Accordingly, while the current implementation focuses on vacuum-based cleaning for clarity and tractability, the underlying planning framework is extensible to other sanitization modalities, and adapting it to sterilization-aware CPP represents a natural and promising direction for future research.

**Author action:** We updated the manuscript by:

- Added sentences in Section 4 Proposed Complete Coverage Path Planning 334 Framework for Aircraft Cabin Cleaning from line 341 to line 344 to state an explicit statement clarifying that the current experiments assume a vacuum-based cleaning mechanism and do not model sterilization-specific constraints.

In the present study, the cleaning task is modeled under the assumption of a vacuum-based mechanism, and sterilization-specific constraints such as UV exposure dosage or spray dispersion are not explicitly considered.

- Added sentences in Section 6.5 Mechanism-Aware Coverage and Integrated Sterilization from line 816 to line 824 to state a paragraph discussing how UV and spray-based sterilization constraints (e.g., dosage, obstruction, safety) could be integrated by extending waypoint generation and cost-function terms.

**Reviewer #2 Point12:** Maps for both aircraft models are sourced from manuals/layout diagrams. However, real cabins exhibit detailed variations (carpets/thresholds/metal rails). Are these modeled in simulations? If not, could conclusions be over-extended?

**Author response:** Thank you for your comment.

In the present study, the cabin maps for both aircraft models are constructed based on official layout diagrams and geometric specifications, which accurately capture the macro-level structure of the cabin, including seat arrangement, aisle width, and under-seat clearance. However, micro-scale surface variations, such as carpet texture, floor thresholds, metal seat rails, and minor elevation changes, are not explicitly modeled in the simulation.

The objective of this work is to evaluate the planning-level feasibility, structure, and scalability of the proposed two-stage coverage framework, rather than to simulate execution-level interaction with detailed cabin surfaces. As such, the conclusions drawn in this paper are limited to the path planning and task allocation level, and do not claim direct performance guarantees under all real-world surface variations.

We agree that incorporating fine-grained surface details and execution-level effects would be necessary for deployment-ready validation. Importantly, the proposed framework does not rely on surface-specific assumptions and can be integrated with higher-fidelity maps or execution-level controllers. Therefore, while the current conclusions are intentionally scoped to planning behavior, extending the simulation to include detailed cabin surface variations represents a valuable direction for future work.

**Author action:** We updated the manuscript by:

- Added sentences in Section 4.4.1 Simulation Environment from line 522 to line 526 to state a statement clarifying that the simulation models macro-level cabin geometry derived from layout diagrams. Explicitly stated that micro-scale surface variations (e.g., carpets, thresholds, metal rails) are not modeled.
- Added sentences in Section 5.2 Modeling Assumptions and Limitations from line 720 to line 738 to state a paragraph explicitly limiting the conclusions to planning feasibility and scalability, and identifying execution-level validation with detailed cabin surfaces as future work.
